# Effect of having private health insurance on the use of health care services: the case of Spain

**DOI:** 10.1186/s12913-017-2667-4

**Published:** 2017-11-13

**Authors:** David Cantarero-Prieto, Marta Pascual-Sáez, Noelia Gonzalez-Prieto

**Affiliations:** Department of Economics, Faculty of Economics and Business, University of Cantabria (Spain), Avenue Los Castros, s/n, 39005 Santander, CP Spain

**Keywords:** Health care utilization, Matching techniques, ECHP, SNHS, EU-SILC

## Abstract

**Background:**

Several stakeholders have undertaken initiatives to propose solutions towards a more sustainable health system and Spain, as an example of a European country affected by austerity measures, is looking for ways to cut healthcare budgets.

**Methods:**

The aim of this paper is to study the effect of private health insurance on health care utilization using the latest micro-data from the European Community Household Panel (ECHP), the Spanish National Health Survey (SNHS) and the European Union Statistics on Income and Living Conditions (EU-SILC). We use matching techniques based on propensity score methods: single match, four matches, bias-adjustment and allowing for heteroskedasticity.

**Results:**

The results demonstrate that people with a private health insurance, use the public health system less than individuals without double health insurance coverage.

**Conclusions:**

Our conclusions are useful when policy makers design public-private partnership policies.

## Background

In recent years, and because of the economic crisis, Spain’s government has been looking for ways to cut healthcare budgets. The main goals are focused on reducing health care expenditure, although patients have to face long waiting lists for hospital admission, lack of medical staff, greater co-payments or participation of the patients in the costs according to the utilization of the services in order to raise efficiency in health care provision.

In Spain, the right to health protection and care is laid out in article 43 of the 1978 Constitution. Also, it is important to point out that the process of devolution of health services available to the Autonomous Communities that had begun in 1981, concluded in 2001 (for further information of the Spanish National Health System, see the formal description made by the Ministry of Health and Consumer Affairs [1]). In this regard, the Spanish National Health System is organized in two levels: “Primary Health Care” and “Specialist Care”. However, access is gained by referral from Primary Health Care (“gatekeeping system”).

The fast growth of expenditure on health care and its relationship with health outcomes have been largely studied in the European Union countries [2–4]. Nowadays, the increase in health expenditure is considered to a sign of a richer society looking for more health care. Part of this increase is because of population aging and technological improvements. In general, access to some level of health care services in European countries is universal for all individuals however they may opt for private health insurance by taking out supplementary coverage. Obviously, health care systems in European countries differ in the source of financing, coverage and means of delivering benefits, but they are also mainly financed through taxation or contributions from employers and employees. This fact justifies the differences between public and private health expenditure.

As a result, over the last few years, there has been a vast amount of literature focus on health care financing and expenditure. In fact, different stakeholders have also undertaken initiatives to propose solutions towards a more sustainable health system. These are carried out specially on studies analysing supplementary private health insurance. Obviously, the expansion (or reduction) of double health insurance coverage has important effects on health care utilization, spending and new technologies [5, 6].

Bago d’Uva and Jones [7] conducted a study on health in several European countries to study the differences in the demand for health. They used the full dataset of the ECHP, which lasted from 1994 until 2001. Using the same dataset, Gonzalez and Clavero [8] concluded that the majority of differences in the number of visits to a general practitioner are explained by the individual characteristics of those insured, while the divergences in the number of consultations with specialists are the result of the overuse of this care by the population with double health insurance coverage. Concerning visits to the general practitioner, that inequality, in favor of those protected by the public system is explained by the endowments of that group and furthermore to an underutilization of this service on the part of individuals who have double coverage. In consultations with specialists, the inequality in favour of the latter group is due to their overuse of the service. Ayala and Rodríguez [9] tested whether participation in work-related activities yields positive results in terms of health outcomes and behaviors in Spain.

From another perspective, Urbanos-Garrido and López-Valcarcel [10] estimated the effect of unemployment on the overall and mental health of the Spanish working-age population. They apply matching techniques to cross-sectional micro data for the Spanish Health Survey concluding that the effect is particularly high on the long-term unemployed. In fact, within a National Health Insurance System, individuals who take out private insurance are likely to be those who anticipate, based on private information, a higher than average demand for health care [11, 12].

Barros et al. [13] studied the effect of extra health insurance on the number of clinical visits. The case under study is Portugal where there is a National Health System, but the civil servants and their dependents have another health insurance. The authors study whether having double health coverage implies that such persons demand more health services than those who only have one. The methodology that they use is matching estimators to estimate the effect on the number of visits to the doctor if they have extra health insurance. In particular, they show the results based on simple matching and biased adjusted matching. They demonstrated that the effect of an extra health insurance is positive and substantial. This effect is more important for the youngest cohort.

The aim of this paper is to study the effect of private health insurance on health care utilization in Spain using the latest micro-data from the European Community Household Panel (ECHP), the Spanish National Health Survey (SNHS) and the European Union Statistics on Income and Living Conditions (EU-SILC). We also combine SNHS and EU-SILC because of limitations of both for the purpose of this study. Following the methodology proposed by Arellano and Meghir [14], we provide statistical evidence on the compatibility of the two samples. Once we have combined both datasets, we study whether having an extra health insurance policy affects the number of times that health care is required. To achieve this aim, we use matching techniques based on propensity score methods. So, we are going to study the effect on an individual’s use of healthcare when he or she has purchased health insurance. Therefore, the problem is to identify the effect of a “treatment”. In this sense, the causal effect of interest is the difference between the outcome with and without treatment. Obviously, an individual cannot be observed in these two situations at the same time.

This paper uses policy evaluation techniques, namely propensity score matching, to assess the extent to which Spanish individuals with double health insurance coverage use (general practitioner and specialist visits) more or less health care than their counterparts who do not have such coverage. Also, we derive the empirical results and discuss our main findings as well as policy implications.

## Methods

### Relevance of double health insurance coverage

In Spain the National Health Service offers universal coverage as a constitutionally-guaranteed right. Nowadays, there are important problems such as the need to control health spending growth, waiting lists, etc. It is, therefore, necessary to evaluate policies with respect to the measures taken to address these problems. One of the solutions that has arisen to reduce both costs and waiting lists is to use and finance private healthcare. However, having double health insurance coverage may increase the number of medical visits. In this case, we state that there is moral hazard [15, 16]. However, there are two types of moral hazard. When the individual changes his/her behavior towards risk because it has extra insurance this is termed ex-ante moral hazard. And the other possibility is that people change their behavior because they have an extra insurance; they seek medical advice in circumstances where if they did not have that extra insurance policy they would not. This may be another problem which has to be avoided to control health care expenditure, so it is necessary to study the behavior of individuals with private health insurance. Thus, comparing the effect of private and public insurance on health care utilization is important, as they complement each other, and also offers insight to policy makers on the relevance of either insurance scheme. Also, the use of micro-data from varied sources to arrive at a conclusion is important.

The use of health care services depends on the type of insurance [17]. In fact, there is a positive effect of private insurance on hospital in-patient services [18]. In Portugal, for example, the effects of health insurance on the number of clinical visits are substantial and positive [13]. However, double coverage creates additional utilization of health care across the whole outcome distribution [19]. In addition, private insurance in France has a strong and significant effect on health care utilization [20]. Besides, there are studies that compare the effect of voluntary private health insurance among different countries, using the out of pocket healthcare spending as outcome. The results indicate that private insurance is a strong incentive to spend more out of pocket healthcare in Spain, Italy, Austria and Denmark [21]. In the case of Spain, using data from the National Health Survey of 1997, people with only public insurance go 2.8 times to the general practitioner for each time that they visit a specialist; individuals with double coverage have a ratio of general practitioner/specialist visits equal to 1.4 [22]. In the case of Catalonia (a Spanish Autonomous Community), there exists a positive effect of double coverage on visits to specialists among non-heads of household [12]. From another point of view, individuals with prescription drug insurance also make more visits to the General Practitioner (GP) than those who do not have that insurance [23]. Other studies analyze the effect of co-payment rates and conclude that a decrease in co-payment rates produces an increase in the demand for health care services [24, 25]. In fact, voluntary health insurance provides complementary cover for services excluded or not fully covered by the state as well as faster access and enlarged consumer choice.

In addition, due to the economic crisis that has existed since 2008, reducing health expenditure and waiting lists is one of the greatest issues of importance for Spain. In fact, unemployment has a negative effect on both Self-Assessed Health (SAH) and mental health [26]. This effect is particularly high for the long-term unemployed. It should be taken account that this crisis has produced a decrease in public health expenditure by 7.2% in 2009 to 6.8% in 2011 [10]. However, private health expenditure has increased from 2.4 to 2.5% in the same period of time. Thus, the study of the potential reduction in health care utilization associated with private insurance is a point of great interest not only for policy makers but also for the whole population.

### Estimation techniques

In this study, we are interested in calculating the effect of double health insurance coverage on health care utilization. In particular, we want to study whether individuals behave differently precisely because they have a private health insurance. This is known as the average treatment effect on treated. To estimate it, we applied matching and propensity score methods that are based on comparing two groups. On the one hand, in the first group, there are individuals who have received treatment and in the second one, called the control group, there are those individuals who have not received treatment but have similar characteristics to those who received it. In particular, each individual from the first group is paired with one or more individuals in the control group. Let the variable *w* be a binary treatment indicator, where *w* = 1 denotes treatment and *w* = 0 otherwise. We have a random vector (*y*_0_, *y*_1_, *w*) from an individual of the population of interest. Thus, the Average Treatment Effect (ATE) on treated is defined as [27]:

1 $$ {ATE}_1=E\left({y}_1-{y}_0|w=1\right)=E\left({y}_1|w=1\right)-E\left({y}_0|w=1\right) $$

where *Y*_0_ and *Y*_1_ represent health outcomes for individuals who do not have private health insurance or those who do, respectively..

We are going to define the causal effect in terms of potential outcomes or counterfactuals [28]. We consider an individual *i*. He or she can receive the treatment and his/her outcome is *y*_1_. If he/she does not receive the treatment, then his/her outcome is *y*_0_. Obviously, an individual cannot be in the two categories. Therefore, we cannot observe both.

Thus, causal effects are comparisons of *y*_0_ and *y*_1_, for example *y*_1_ − *y*_0_ or *y*_1_/*y*_0_ [27]. We will focus on measuring *y*_1_ − *y*_0_. For this, we need to make an additional assumption: We have an independent, identically distributed sample from the population. This implies that the treatment on individual *i* affects only to individual *i*, which is called the Stable Unit Treatment Value Assumption (SUTVA). In most programs the individual is the one who decides whether to participate. Thus there is self-selection into treatment. If we assume that *w* is independent of *y*_0_, we can estimate *ATE*_1_ consistently:

2 $$ {\displaystyle \begin{array}{l}E\left(y|w=1\right)-E\left(y|w=0\right)=E\left({y}_0|w=1\right)-E\left({y}_0|w=0\right)+\\ {}+E\left({y}_1-{y}_0|w=1\right)=\left[E\left({y}_0|w=1\right)-E\left({y}_0|w=0\right)\right]+{ATE}_1\end{array}} $$

If it holds that

3 $$ E\left({y}_0|w\right)=E\left({y}_0\right), $$

substituting it in eq. (2) we have an unbiased estimator of *ATE*_1_.

When *w* and (*y*_0_, *y*_1_) are allowed to be correlated we need the assumptions that Rosenbaum y Rubin proposed [27] and which were called ignorability of treatment:

*Assumption 1*: Conditional on *x*, *w* and (*y*_0_, *y*_1_) are independent.

Often it is enough to assume:

*Assumption 2*: a) *E*(*y*_0_| *x*, *w*) = *E*(*y*_0_| *x*) and b) *E*(*y*_1_| *x*, *w*) = *E*(*y*_1_| *x*).

Under *Assumption 2* the average treatment effect conditional on *x* (*ATE*(*x*)) and the average treatment effect of the treated conditional on *x* (*ATE*_1_(*x*)), are identical. To estimate *ATE*_1_ we can use regression methods that can be nonparametric and parametric. As we have a random sample on (*y*, *w*, *x*) from the population, *r*_1_(*x*) ≡ *E*(*y*| *x*, *w* = 1) and *r*_0_(*x*) ≡ *E*(*y*| *x*, *w* = 0) are no parametrically identified. They are conditional expectations that depend entirely on observables and they can be consistently estimated.

In consequence, to apply matching methods we need to accept *Assumption 1*, which is a particular case of a balancing score. A balancing score is a function *b*(*x*) of the observed covariates such that (*y*_0_, *y*_1_ ⊥ *w*) ∣ *b*(*x*).

Thus, the simplest case of balancing score is *b*(*x*) = *x*. To ensure compliance of *Assumption 1*, the vector of covariates *x* should contain all information affecting the participation in the program and the variable that is being studied. One of the balancing scores most used is the propensity score defined by Rosenbaum and Rubin [27]. They demonstrated that if treatment assignment is strongly ignorable, conditioning on the propensity score allows one to obtain unbiased estimates of average treatment effects.

Hence, a key point is to calculate the corresponding propensity score. Let *x* be a set of covariates. The propensity score is the conditional probability of assignment to treatment, given the covariates. We denote it as:

4 $$ p(x)\equiv P\left(w=1|x\right). $$

Once we have calculated the propensity score, we have several methods for applying matching techniques. In particular, we have used nearest-neighbor matching [29]. This method will match the individuals whose propensity score shows the smallest difference. Let *T* be the set of treated units and *C* the set of control units. Let *C*(*i*) be the set of control units matched to the treated unit *i* with an estimated value of the propensity score of *p*_*i*_, nearest-neighbor matching sets:

5 $$ C(i)=\underset{j}{\min}\left\Vert {p}_i-{p}_j\right\Vert . $$

In addition, to test the sensitivity of our results, we have considered different estimators: one to-one propensity score matching, using 4 matches, using 4 matches and bias adjustment and finally allowing for heteroskedasticity.

### Data description and exposure variables

Therefore, the idea of our empirical approach is as follows. Firstly, the data used in this paper are obtained from the ECHP. This survey contains data on individuals and households for the European Union countries with eight waves available (1994 to 2001). The main advantage is that information is homogeneous among countries since the questionnaire is similar throughout them. This source of data is coordinated by the Statistical Office of the European Communities (EUROSTAT). Also, this survey includes rich new information about income, education, employment, health, etc. This representative survey of households in different European Union countries was carried out for the first time in 1994 and 60,500 households were interviewed (approximately 170,000 individuals).

In order to determine the main socio-demographic characteristics of people who have a private health insurance, we have classified them into six groups of variables: personal and household characteristics, education level, marital status, personal earnings, occupational status and variables related to individuals’ health. Definition of all the variables and the basic descriptive statistics are shown in Table 1.

**Table 1:** Variable definitions and descriptive statistics using the ECHP

		2001		2000		1999		1998	
Name	Definition	Mean	Std. Dev.	Mean	Std. Dev.	Mean	Std. Dev.	Mean	Std. Dev.
Personal Characteristics									
FEMALE	1 if female, 0 otherwise	0.5202	0.4996	0.5193	0.4996	0.5198	0.4996	0.5181	0.4997
AGE	Individual’s age	46.2874	19.6670	45.9765	19.6249	45.6354	19.5239	45.2911	19.4838
Education									
EDUC1	1 if less than secondary level (ISCED 0–2), 0 otherwise	0.4273	0.4947	0.4378	0.4961	0.4399	0.4964	0.4417	0.4966
EDUC2	1 if third level education (ISCED 5–7)	0.1319	0.3384	0.1278	0.3339	0.1211	0.3263	0.1161	0.3203
Marital Status									
SINGLE	1 if single, 0 otherwise	0.3001	0.4583	0.3032	0.4596	0.3071	0.4613	0.3088	0.4620
SEPARATED	1 if separated, 0 otherwise	0.0144	0.1190	0.0152	0.1223	0.0150	0.1214	0.0150	0.1216
DIVORCED	1 if divorced, 0 otherwise	0.0097	0.0980	0.0095	0.0970	0.0085	0.0921	0.0081	0.0894
WIDOW	1 if widowed, 0 otherwise	0.0893	0.2851	0.0891	0.2848	0.0857	0.2799	0.0867	0.2814
MARRIED	1 if married, 0 otherwise	0.5866	0.4925	0.5831	0.4931	0.5837	0.4930	0.5814	0.4933
Personal Earnings									
LOGWAGE	Natural logarithm of the individual’s earnings	8.5711	1.5986	8.4468	1.7219	8.4794	1.5669	8.3946	1.5878
Employment									
UNEMPLOYMENT	1 if unemployed, 0 otherwise	0.0605	0.2385	0.0640	0.2447	0.0709	0.2567	0.0855	0.2796
Health Status									
SMOKE	1 if individual is a smoker, 0 otherwise	0.3232	0.4677	0.3245	0.4682	0.3370	0.4727	0.3475	0.4762
NUMBER_VISITS1	Number of visits to general practitioner	4.0828	6.9875	3.5232	5.6475	3.6494	6.1505	3.8179	6.6565
NUMBER_VISITS2	Number of visits to specialist doctors in the previous year	1.7091	4.0508	1.5690	3.4261	1.5577	3.7921	1.6237	3.7099
HOSPITAL	1 if individual has been hospitalized in the previous year, 0 otherwise	0.0871	0.2819	0.0792	0.2700	0.0789	0.2696	0.0828	0.2756
FAIR_HEALTH	1 if individual’s self-assessed health is fair, 0 otherwise	0.2178	0.4127	0.2018	0.4014	0.2057	0.4043	0.2082	0.4060
BAD_HEALTH	1 if individual’s self-assessed health is bad or very bad, 0 otherwise	0.1056	0.3074	0.1099	0.3128	0.1024	0.3032	0.1164	0.3207
CHRONIC	1 if individual is an chronic sick, 0 otherwise	0.2301	0.4209	0.2144	0.4104	0.2196	0.4140	0.2369	0.4252
PRIVATE_INSURANCE	1 if individual has private insurance, 0 otherwise	0.1157	0.3199	0.0970	0.2960	0.0980	0.2973	0.1003	0.3004

Source: Authors’ elaboration

As personal characteristics we have included two variables: the individual’s age (in years) and gender (building a dummy variable which takes value of 1 if individual is female and 0 otherwise). To allow for a flexible relationship between the probability of having private health insurance and AGE, a quadratic polynomial function of this variable is included (AGE2 = Age^2). The second group of variables refers to the maximum level of education completed. In the ECHP, education is classified into three categories based on ISCED classification: lower than secondary level (ISCED 0–2), second stage of secondary level (ISCED 3) and third level (ISCED 5–7). Thus, two dummy variables have been included: bellow secondary level (EDUC1) and tertiary level education (EDUC2). Thirdly, regarding marital status, we have considered four variables (SINGLE, SEPARATED, DIVORCED and WIDOWED) with married as the reference category. On the other hand, we are concerned with the influence of income on having private health insurance. Our income variable is a natural logarithm of the individual’s wage (LOGWAGE). Other variables included in the analysis related to the labor market are employment status. We have considered a dummy variable that takes value one if the individual is unemployed and zero otherwise (UNEMPLOYMENT). Also, we have considered other variables related to health status. We have taken into account whether an individual has any chronic condition (CHRONIC), whether the individual has been in hospital the previous year (HOSPITAL), the number of visits to the doctor (NUMBER_VISITS) and finally we have considered individuals’ SAH. We have defined two dummy variables: FAIR_HEALTH (1 if individual’s SAH is fair and 0 otherwise) and BAD_HEALTH (1 if individual’s SAH is bad or very bad and 0 otherwise). Moreover, we have incorporated another dummy variable which takes value 1 if the individual smokes daily or occasionally (SMOKER). Finally, we have defined another dummy variable that indicates whether the individual has private health insurance (PRIVATE_INSURANCE).

The effect of double health insurance coverage has been researched using information from another two independent sources: the European Union Statistics on Income and Living Conditions (EU-SILC, 2011 and 2012) and the Spanish National Health Survey (SNHS) (2011/2012). The EU-SILC contains data on individuals and households for European Union countries. It is published annually and the main advantage is that information is homogeneous among countries since the questionnaire is similar throughout them and coordinated by EUROSTAT. Also, this survey includes rich new information about income, education, employment, health, etc. On the other hand, the SNHS provides general information on the health of the Spanish population in order to plan and evaluate interventions in health. The 2011–2012 survey consists of approximately 24,000 dwellings and it includes information on assessment of general, physical and mental health, and it identifies the main problems that citizens feel (chronic diseases, ailments, accidents, limitations to performance of activities of daily living, etc.). The two surveys are complementary in the way that they both contain demographic characteristics, education, health status, etc. In fact, the definition of most of the variables in the two questionnaires is similar but only the EU-SILC contains information about income and only the SNHS includes information about the number of visits to general practitioners or specialist doctors, type of health insurance and lifestyle characteristics. Thus, income information is obtained from EU-SILC, while information of health status is obtained from SNHS. Obviously, both surveys refer to the same period of time.

In order to establish the main socio-demographic characteristics of people who have a private health insurance, we have classified them into four groups of variables: personal characteristics, marital status, variables related to individuals’ health and income. Table 2 shows explanatory variables used in estimations and their corresponding definitions. Firstly, as personal characteristics we have included two variables: the individual’s age (in years) and sex (building a dummy variable which takes value of 1 if individual is male and 0 otherwise). To allow for a flexible relationship between the probability of having a private health insurance and AGE, a quadratic polynomial function of this variable is included (AGE^2^). Secondly, regarding marital status, we have considered three variables (SINGLE, SEPARATED_DIVORCED and WIDOWED) with married as the reference category.

**Table 2:** Variable names and descriptive statistics using the EU-SILC (2011 and 2012) and the SNHS (2011/12)

Variable	Variable description	EU-SILC (2011)		EU-SILC (2012)		SNHS (2011/2012)	
		Mean	SD	Mean	SD	Mean	SD
MALE	1 if male, 0 otherwise	0.48	0.50	0.48	0.50	0.46	0.5
AGE	Individual’s age (years)	49.66	18.7	50.04	18.76	51.6	19.09
SINGLE	1 if single, 0 otherwise	0.29	0.45	0.30	0.46	0.28	0.45
SEPARATED_DIVORCED	1 if separated or divorced	0.05	0.21	0.05	0.21	0.06	0.25
WIDOW	1 if widow, 0 otherwise	0.08	0.28	0.09	0.28	0.13	0.34
MARRIED	1 if married, 0 otherwise	0.58	0.49	0.57	0.50	0.52	0.50
EARNINGS	Individual’s earnings	6445.47	9962.39	6147.71	9746.15	–	–
SMOKE	1 if smoker, 0 otherwise	–	–	–	–	0.25	0.43
DRINK	1 if consumes alcohol regularly, 0 otherwise	–	–	–	–	0.49	0.50
OBESE	1 if obese, 0 otherwise	–	–	–	–	0.39	0.49
CHRONIC	1 if chronic condition, 0 otherwise	0.25	0.44	0.28	0.45	0.47	0.50
LIMIT	1 if, individual has a limitation, 0 otherwise	0.23	0.42	0.23	0.42	0.22	0.42
GOOD_HEALTH	1 if good or very good SAH, 0 otherwise	0.73	0.44	0.72	0.45	0.68	0.47
BAD_HEALTH	1 if bad or very bad SAH, 0 otherwise	0.08	0.27	0.09	0.28	0.09	0.29
NUMBER_VISITS_GENERAL	Number of visits to general practitioner in last 4 weeks	–	–	–	–	1.31	0.87
NUMBER_VISITS_SPECIALIST	Number of visits to specialist doctor in last 4 weeks	–	–	–	–	1.33	1.08
PRIVATE_INSURANCE_INDIVIDUAL	1 if individual has private insurance taken out by him, 0 otherwise	–	–	–	–	0.09	0.29
PRIVATE_INSURANCE_COMPANY	1 if individual has private insurance taken out by his company, 0 otherwise	–	–	–	–	0.02	0.15

Source: Authors’ elaboration

Also, we have considered different variables related to health status. We have taken into account whether an individual has a chronic condition (CHRONIC) or limitation in his/her life (LIMIT), the number of visits to the general practitioner (NUMBER_VISITS_GENERAL) and the number of visits to the specialist (NUMBER_VISITS_SPECIALIST). Also, we have considered individuals’ Self-Assessed Health (SAH) and we have defined two dummy variables: GOOD_HEALTH (1 if individual’s SAH is good or very good, 0 otherwise) and BAD_HEALTH (1 if individual’s SAH is bad or very bad, 0 otherwise), leaving fair health as the reference category. In addition, we have incorporated another dummy variable which takes value 1 if individual smokes daily or occasionally (SMOKER). In addition, DRINK and OBESE are two dummy variables. They indicate whether the individual consumes alcohol regularly and whether he/she is obese (Body Mass Index greater than 30), respectively. Besides, we have defined two dummy variables that indicate whether the individual has private health insurance taken out by him/herself (PRIVATE_INSURANCE_INDIVIDUAL) or by the company (PRIVATE_INSURANCE_COMPANY).

Finally, as pointed out before, we are concerned with the influence of income on having a private health insurance. The problem is that the SNHS does not contain information about income. For this reason, this information has to be obtained from the EU-SILC. It is worth noting that the composition of both samples does not differ very much.

Also, the data do make clear that those individuals who have private health insurance (taken out by themselves or their company), use public health system less than individuals without double health insurance coverage. In this sense, it is important to notice that in Spain, it is not usual to have at the same time individual private insurance and private insurance taken out by the company. In fact, in our surveys, this option is not considered. In Table 3, we can see that 67.21% of individuals who have private health insurance taken out by themselves went to a public general practitioner on their last visit (41.79% to a specialist doctor), while those percentages are 95.98 and 88.82% for individuals who do not have private health insurance. Moreover, the behavior is similar when the private health insurance is taken out by the company. As Table 3 shows, 76.99% of individuals who have private health insurance taken out by the company went to a public general practitioner on their last visit (41.30% to a specialist doctor) while those percentages are 94.13 and 83.22% for individuals who do not have such insurance.

**Table 3:** Level of healthcare services utilization by type of health coverage and functional dependence of the doctor

Private insurance taken out by the individual	Functional dependence	General practitioner	Specialist
		Percentage	Percentage
Yes	Public Health	67.21	41.79
	Medical society	11.89	21.21
	Private Consultation	18.24	35.55
	Others	2.66	1.46
No	Public Health	95.98	88.82
	Medical society	1.31	3.1
	Private Consultation	1.92	6.86
	Others	0.78	1.22
Private insurance taken out by the company	Functional dependence	General practitioner	Specialist
		Percentage	Percentage
Yes	Public Health	76.99	41.30
	Medical society	7.96	19.57
	Private Consultation	11.50	27.17
	Others	3.54	11.96
No	Public Health	94.13	83.22
	Medical society	2.00	5.31
	Private Consultation	3.00	10.52
	Others	0.87	0.95
No Private insurance	Functional dependence	General practitioner	Specialist
		Percentage	Percentage
	Public Health	100	74,90
	Medical society	0	0,91
	Private Consultation	0	23,78
	Others	0	0,41

Source: Author’s elaboration based on SNHS (2011/2012)

## Results

We focus on those characteristics which could explain an individual having private health insurance [8]. A set of factors gathered in a vector *x* explain this fact so the probability model is a regression:

6 $$ E\left(y|x\right)=F\left(x,\beta \right). $$

The set of parameters *β* reflects the effect of changes in *x* on the probability. In order to estimate this equation, a nonlinear specification of *F*(.) can avoid logical inconsistency and the possibility of predicted probabilities outside the range [0, 1]. The most common nonlinear parametric specifications are logit and probit models which have been analysed, and we use a latent variable interpretation.

Table 4 shows the results of the probit equation for the years 1998 to 2001. The aim is to model the probability of an individual having private health insurance as a function of socioeconomic characteristics. To interpret the quantitative implications of these findings, we compute average and partial effects. According to this, results seem to be similar every year. The unemployment coefficient is always negative. So, as expected, an unemployed person is less likely to have private health insurance than a person who is working. On the other hand EDUC2 coefficient is positive, which confirms that the level of education is highly correlated with double health insurance coverage.

**Table 4:** Probit Estimates

	2001					2000				
	Coef.	Std. Err.	z	P > |z|	dF/dx	Coef.	Std. Err.	z	P > |z|	dF/dx
AGE	0.0053	0.0014	3.7900	0.0000	0.0009	0.0069	0.0015	4.6500	0.0000	0.0010
FEMALE	0.0048	0.0373	0.1300	0.8970	0.0008	0.0620	0.0394	1.5700	0.1160	0.0093
UNEMPLOYMENT	−0.4790	0.0967	−4.9600	0.0000	−0.0622	−0.3185	0.0919	−3.4600	0.0010	−0.0389
WAGE	0.0235	0.0122	1.9200	0.0550	0.0040	0.0353	0.0125	2.8200	0.0050	0.0053
EDUC1	−0.6691	0.0547	−12.2400	0.0000	−0.1094	−0.6647	0.0570	−11.6600	0.0000	−0.0958
EDUC2	0.4817	0.0451	10.6700	0.0000	0.1022	0.4790	0.0473	10.1200	0.0000	0.0902
FAIR_HEALTH	−0.0769	0.0517	−1.4900	0.1370	−0.0129	−0.1284	0.0568	−2.2600	0.0240	−0.0182
BAD_HEALTH	−0.3872	0.0899	−4.3100	0.0000	−0.0546	−0.3551	0.0905	−3.9300	0.0000	−0.0438
CHRONIC	0.0234	0.0553	0.4200	0.6730	0.0041	0.1204	0.0582	2.0700	0.0390	0.0188
HOSPITAL	0.1440	0.0648	2.2200	0.0260	0.0267	0.1489	0.0693	2.1500	0.0320	0.0242
SMOKE	−0.0509	0.0386	−1.3200	0.1880	−0.0087	0.0041	0.0403	0.1000	0.9180	0.0006
MARRIED	0.1444	0.0381	3.7900	0.0000	0.0245	0.1716	0.0401	4.2800	0.0000	0.0252
_cons	−1.5293	0.1110	−13.7800	0.0000		−1.8762	0.1144	−16.4000	0.0000	
Numb. of obs	9507.0000					9558.0000				
Log likelihood	−3134.2760					−2798.6910				
Pseudo R2	0.0927					0.0904				
	1999					1998				
	Coef.	Std. Err.	z	P > |z|	dF/dx	Coef.	Std. Err.	z	P > |z|	dF/dx
AGE	0.0065	0.0014	4.5200	0.0000	0.0010	0.0079	0.0014	5.6600	0.0000	0.0013
FEMALE	0.0598	0.0388	1.5400	0.1230	0.0093	0.0364	0.0374	0.9700	0.3300	0.0060
UNEMPLOYMENT	−0.3352	0.0892	−3.7600	0.0000	−0.0423	−0.2565	0.0750	−3.4200	0.0010	−0.0363
WAGE	0.0433	0.0135	3.1900	0.0010	0.0067	0.0145	0.0121	1.2000	0.2300	0.0024
EDUC1	−0.6338	0.0560	−11.3300	0.0000	−0.0950	−0.6165	0.0529	−11.6400	0.0000	−0.0983
EDUC2	0.4106	0.0478	8.5900	0.0000	0.0775	0.4479	0.0473	9.4700	0.0000	0.0907
FAIR_HEALTH	−0.1227	0.0552	−2.2200	0.0260	−0.0181	−0.1618	0.0524	−3.0900	0.0020	−0.0249
BAD_HEALTH	−0.4180	0.0925	−4.5200	0.0000	−0.0516	−0.3484	0.0821	−4.2400	0.0000	−0.0478
CHRONIC	0.0597	0.0574	1.0400	0.2980	0.0094	0.0406	0.0539	0.7500	0.4520	0.0067
HOSPITAL	0.2436	0.0666	3.6600	0.0000	0.0432	0.0447	0.0660	0.6800	0.4980	0.0075
SMOKE	−0.0487	0.0398	−1.2200	0.2210	−0.0075	−0.0216	0.0382	−0.5700	0.5710	−0.0035
MARRIED	0.1271	0.0393	3.2300	0.0010	0.0194	0.0910	0.0381	2.3900	0.0170	0.0148
_cons	−1.8484	0.1200	−15.4000	0.0000		−1.5825	0.1056	−14.9900	0.0000	
Numb. of obs	9619.0000					9934.0000				
Log likelihood	−2899.6812					−3116.5375				
Pseudo R2	0.0830					0.0744				

(*) dF/dx is for discrete change of dummy variable from 0 to 1. z and P > |z| are the test of the underlying coefficient being 0

Source: Author’s elaboration from ECHP

Moreover, we want to analyse the effect of double health insurance coverage on health care utilization. In particular, we wish to study whether individuals behave differently precisely because they have private health insurance. We are going to estimate the average treatment effect and the average treatment effect on those treated. To calculate the average treatment effect on those treated we have used four different matching models: single match, four matches, bias-adjustment and allowing for heteroskedasticity. Table 5 shows the results based on the ECHP. The first estimator that we have considered in row one is the One to One propensity score matching. We find that the difference between the matched treated and the matched controls is −0.2779 in 2001, −0.1418 in 2000, −0.1235 in 1999 and −0.6379 in 1998 while the *Z*-statistics for *H*_0_ are −1.22, −0.72, −0.54 and −2.83, respectively for *ATE*_1_ on visits to the general practitioner. On the other hand, we find that when we analyse *ATE*_1_ on visits to a specialist, the difference between the matched treated and the matched controls is 0.7933 in 2001, 0.6691 in 2000, 1.0283 in 1999 and 0.6900 in 1998. By using four matches, results are quite similar either for visits to a general practitioner or visits to a specialist. We choose it because in this way we do not rely on too few information-matching observations that are not sufficiently similar.

**Table 5:** Matching and regression estimates of the effect of private health insurance on general practitioner and specialist visits

2001	Visits to general practitioner				Visits to specialists			
	*ATE*_*1*_	Std. Err.	Z	P > |z|	ATE_*1*_	Std. Err.	t	P > |z
*m* = 1	−0.2779	0.2279	−1.22	0.223	0.7933	0.2070	3.83	0.000
*m* = 4	−0.3132	0.1668	−1.88	0.060	0.7710	0.1569	4.91	0.000
*m* = 4 and bias-adjustment	−0.3207	0.1668	−1.92	0.055	0.7738	0.1569	4.93	0.000
*m* = 4 and allowing for heterokedasticity	−0.3207	0.1618	−1.98	0.048	0.7738	0.1696	4.56	0.000
*n*	9557				9558			
2000	Visits to general practitioner				Visits to specialists			
	*ATE*_*1*_	Std. Err.	Z	P > |z|	ATE_*1*_	Std. Err.	t	P > |z
*m* = 1	−0.1418	0.1977	−0.72	0.473	0.6691	0.1597	4.19	0.000
*m* = 4	−0.2907	0.1651	−1.76	0.078	0.5844	0.1314	4.45	0.000
*m* = 4 and bias-adjustment	−0.2917	0.1650	−1.77	0.077	0.5850	0.1314	4.45	0.000
*m* = 4 and allowing for heterokedasticity	−0.2917	0.1499	−1.50	0.052	0.5850	0.1383	4.23	0.000
*n*	9745							
1999	Visits to general practitioner				Visits to specialists			
	*ATE*_*1*_	Std. Err.	Z	P > |z|	ATE_*1*_	Std. Err.	t	P > |z
*m* = 1	−0.1235	0.2272	−0.54	0.587	1.0283	0.1743	5.90	0.000
*m* = 4	−0.1573	0.1795	−0.88	0.381	0.9280	0.1418	6.55	0.000
*m* = 4 and bias-adjustment	−0.1685	0.1793	−0.94	0.347	0.9274	0.1418	6.54	0.000
*m* = 4 and allowing for heterokedasticity	−0.1685	0.1821	−0.93	0.355	0.9274	0.1294	7.17	0.000
*n*	10,008				10,006			
1998	Visits to general practitioner				Visits to specialists			
	*ATE*_*1*_	Std. Err.	Z	P > |z|	ATE_*1*_	Std. Err.	t	P > |z
*m* = 1	−0.6379	0.2257	−2.83	0.005	0.6900	0.1694	4.07	0.000
*m* = 4	−0.5274	0.1794	−2.94	0.003	0.7624	0.1367	5.58	0.000
*m* = 4 and bias-adjustment	−0.5398	0.1792	−3.01	0.003	0.7633	0.1368	5.58	0.000
*m* = 4 and allowing for heterokedasticity	−0.5398	0.1794	−3.01	0.003	0.7633	0.1224	6.24	0.000
*n*	10,249				10,253			

Source: Author’s elaboration from ECHP

For all the specifications at hand, we can conclude that the *ATE*_1_s are significantly different from zero at the 1% level when we are considering the effect of private health insurance on specialist visits whereas the *ATE*_1_s are not always significant when we consider as outcome “Visits to general practitioner”. It depends on the year and the estimator considered. Similar findings are obtained when we use the bias-corrected matching estimator or allowing for heteroskedasticity. It adjusts the difference within the matches for the differences in their covariate values where the last method estimates the standard error allowing for heteroskedasticity. Our results show that when the standard error is estimated under these weaker conditions the estimated *ATE*_1_ is always significant at the 1% level.

As we are interested in expanding this analysis to the next years (note that ECHP is only available till 2001), we have combined EU-SILC and SNHS using the model described by Arellano and Meghir [14]. We have a first sample which is not enough to identify the parameter of interest and a second one which includes information on additional variables and provides the complementary number of variables that are necessary to identify our parameters of interest. For this purpose, we have estimated personal income by using the EU-SILC calculated by the following model:

7 $$ w={\beta}_0+\sum \limits_{k=1}^K{\beta}_k{X}_k+\varepsilon, $$

where *w* corresponds to the logarithm of individual’s earnings, *X*_*k*_ and *ε* are the *k-th* explanatory variable and a random error term, respectively. This methodology is based on regression analysis, and departs from Ordinary Least Squares (OLS) estimation of a logarithmic individual wage equation. Once personal income is estimated, we have allocated it to the SNHS (2011/2012).

Thus, we are going to estimate the effect of having private health insurance (double health insurance coverage) on general practitioner and specialist visits. Table 6 reports the *ATE*_1_ estimates for 2011 and 2012 in the case where private insurance is taken out by individual or by the company, respectively. Also, to test the robustness of our results, we have combined EU-SILC (2011) with SNHS (2011/2012) and have combined EU-SILC (2012) with SNHS (2011/2012). To calculate the average treatment effect on the treated we have used nearest neighbor matching. As can be noticed, double health coverage produces an increase on the number of visits to the specialist doctor and on the number of consultations with the general practitioner on those individuals who have taken out private insurance. The results indicate that for the individuals in our sample, the average effect of having private health insurance (taken out by the individual or by the company) is an increase in the number of consultations with general practitioner and specialist. The estimated *ATE*_1_ is always statistically significant ranging between 0.069 and 0.13 for the visits to the general practitioner and between 0.067 and 0.112 for the visits to specialist doctors.

**Table 6:** *ATE*_*1*_ (*m* = 1) on the number of consultations with General Practitioner and specialists by type of private insurance (taken out by the individual or taken out by the company) using SNHS (2011/2012) and EU-SILC (2011 and 2012)

	2011									
	Visits to General Practitioner					Visits to specialists				
Type of private insurance	N. treat.	N. contr.	*ATE*_*1*_	Std. Err.	t	N. treat.	N. contr.	*ATE*_*1*_	Std. Err.	t
Taken out by the individual	1993	3122	0.131	0.022	6.035	1993	1394	0.109	0.021	5.149
Taken out by the company	508	1239	0.069	0.031	2.224	508	637	0.067	0.036	1.851
Undifferentiated	2501	3163	0.121	0.019	6.232	2501	1402	0.101	0.020	5.073
	2012									
	Visits to General Practitioner					Visits to specialists				
Type of private insurance	N. treat.	N. contr.	*ATE*_*1*_	Std. Err.	t	N. treat.	N. contr.	*ATE*_*1*_	Std. Err.	t
Taken out by the individual	1993	3106	0.130	0.022	5.988	1993	1390	0.112	0.021	5.341
Taken out by the company	508	1231	0.078	0.031	2.485	508	632	0.070	0.036	1.916
Undifferentiated	2501	3166	0.126	0.019	6.503	2501	1405	0.080	0.021	3.790

Source: Author’s elaboration based on SNHS and EU-SILC

**Fig. 1:**
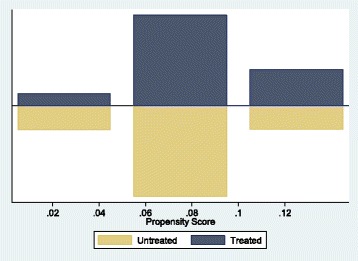
Propensity score histogram by treatment

Obviously, we are assuming that potential outcomes are independent of treatment assignment (un-confoundedness). Therefore, selection is based on observable characteristics and all variables in which influence treatment assignment and potential outcomes are observed simultaneously [30]. However, a further requirement besides independence is the common support or overlap condition. Figure 1 shows the propensity score histogram by treatment status and as can be noticed, we do not have overlap problems. Above the horizontal line is the propensity score histogram of the control group (*w* = 0) and below, the treatment one (*w* = 1). The histogram shows how many treated and control units are matched within each propensity score stratum. As long as there are at least as many untreated units as there are treated units, we can match both using neighbor algorithm. Moreover, another point of interest is the one related with potential endogeneity problems. These issues may arise by the way in which the relevant health status is observed in social surveys. However, we have assumed exogeneity of health indicators based on the results obtained by Urbanos et al. [10], Kreider [31] and Lindeboom et al. [32]. In fact, it is assumed that the effect of taking out private health insurance on health status is a gradual process rather than an instantaneous effect.

## Discussion

This paper attempts to assess the importance of the effect of private health insurance on the use of health care services (visits to General Practitioners and specialist doctors) based on Spanish data. First of all, using the ECHP and applying public evaluation policy techniques, we have studied whether there are differences in the number of visits to specialists (mainly preceded by visits to general practitioners) and general practitioners between individuals with different healthcare coverage through additional affiliation to mutual or private health insurance companies. In this sense, there is no empirical evidence of an overutilization of health care by the population with double health insurance coverage. We have used matching techniques to estimate the average treatment effect on those treated who have private health insurance on the number of medical visits. We replicate a randomized experiment by looking for treated and control groups with similar covariate distributions. This goal has been achieved by choosing well-matched samples of the original treated and control groups, thereby reducing bias due to the covariates.

Besides, we show that using more recent data, in Spain, 12.45% of the population had mixed health care coverage (SNHS, 2011–12). This refers to those persons who have at the same time both public and private health care coverage. Private medical insurance allows individuals to avoid waiting lists and receive fast-track consultations. In order to analyze such “extra-coverage” we rely on propensity score methods. The results of all models are quite similar and they show that the effect of having a private health insurance on the visits to general practitioner on those who have private health insurance is an increase in the number of consultations by 0.069 to 0.13 and its effect on the number of visits in the specialist doctor is a variation of consultations by 0.067 to 0.112. We have also found differences depending on whether the health insurance is taken out by the individual or by the company.

## Conclusions

By using a large data sample, we conclude that having double health insurance coverage has an important effect on health care utilization in Spain which suggests that promoting private health insurance can yield a decrease in waiting lists and public health expenditure even though it could damage the most vulnerable population groups and disadvantaged social classes, enhancing something undesirable: the inequality gap.

Overall, our study has shown that evaluation of public policies is important not only because it provides feedback on the efficiency, effectiveness and performance of public policies but it can also be critical to policy improvement and innovation. Indeed, our results are very useful when policy makers design public-private partnership policies that could benefit the whole population. In this sense, it is important to study whether promoting private medical insurance would reduce waiting lists and increase self-assessed health. In fact, new deductions on taxes could be an alternative to promote private health insurance.

In Spain, as a result of the economic situation, there exists an important problem regarding “long waiting lists” for non-urgent medical care, in diagnostic or therapeutic procedures. In this sense, it is important to study whether promoting private medical insurance would reduce waiting lists and increase self-assessed health [33, 34]. Therefore, evaluation of economic policies provides feedback on their efficiency, effectiveness and performance and can be critical to policy improvement and innovation.

## Abbreviations

ATE: Average treatment effect
ECHP: European Community Household Panel
EUROSTAT: Statistical Office of the European Communities
EU-SILC: European Union Statistics on Income and Living Conditions
OLS: Ordinary least squares
SAH: Self-Assessed Health
SNHS: Spanish National Health Survey
SUTVA: Stable Unit Treatment Value Assumption
